# Discovery and development of trastuzumab deruxtecan and safety management for patients with HER2-positive gastric cancer

**DOI:** 10.1007/s10120-021-01196-3

**Published:** 2021-05-16

**Authors:** Kohei Shitara, Eishi Baba, Kazumasa Fujitani, Eiji Oki, Satoshi Fujii, Kensei Yamaguchi

**Affiliations:** 1 Department of Gastroenterology and Gastrointestinal Oncology, National Cancer Center Hospital East, 6-5-1 Kashiwanoha, Kashiwa, Chiba 277-8577 Japan; 2 Department of Oncology and Social Medicine, Graduate School of Medical Sciences, Kyushu University, 3-1-1 Maidashi, Higashi-ku, Fukuoka, 812-8582 Japan; 3 Department of Surgery, Osaka Prefectural General Medical Centre, 3-1-56 Bandaihigashi, Sumiyoshi-ku, Osaka, 558-8558 Japan; 4 Department of Surgery and Science, Graduate School of Medical Sciences, Kyushu University, 3-1-1 Maidashi, Higashi-ku, Fukuoka, 812-8582 Japan; 5 Department of Molecular Pathology, Yokohama City University Graduate School of Medicine, 3-9 Fukuura, Kanazawa-ku, Yokohama, 236-0004 Japan; 6 Department of Gastroenterological Chemotherapy, The Cancer Institute Hospital of Japanese Foundation for Cancer Research, 3-8-31 Ariake, Koto-ku, Tokyo, Japan

**Keywords:** Gastric cancer, HER-2, Antibody–drug conjugate, Review, Topoisomerase I inhibitor

## Abstract

Approximately 12–15% of gastric cancers (GCs) are human epidermal growth factor receptor-2 (HER2)-positive (HER2 immunohistochemistry 3 + or 2 + /in situ hybridization + [*ERBB2*/*CEP17* ≥ 2.0]). While the anti-HER2 monoclonal antibody trastuzumab, in combination with chemotherapy, is the standard treatment for HER2-positive GC, other HER2-targeted therapies have not demonstrated survival benefits in patients with GC, despite showing efficacy in patients with HER2-positive breast cancer. This indicates that there are unique challenges to the use of currently available HER2-targeted therapies for the treatment of HER2-positive GC. Trastuzumab deruxtecan (T-DXd) is an antibody–drug conjugate consisting of an anti-HER2 human monoclonal IgG1 antibody with the same amino acid sequence as trastuzumab, an enzymatically cleavable peptide-based linker, and DXd, a novel topoisomerase I inhibitor, as its released payload. T-DXd has a high drug–antibody ratio (approximately 8) and a demonstrated bystander antitumor effect. It has demonstrated significant efficacy when compared with standard therapies and is approved as third- or later-line treatment for HER2-positive GC in Japan and second- or later-line treatment in the US. T-DXd treatment is associated with gastrointestinal and hematological adverse events, and a risk of interstitial lung disease (ILD), with the ILD risk being higher in Japan than in countries other than Japan. However, most adverse events, including ILD, can be managed with proactive monitoring and T-DXd dose modification, and initiation of adequate treatment. In this review, we summarize the discovery and development of T-DXd and provide guidance for T-DXd safety management, including ILD monitoring, for patients with HER2-positive GC.

## Introduction

Human epidermal growth factor receptor-2 (HER2) is a member of the epidermal growth factor receptor (EGFR) tyrosine kinase family that is encoded by the gene *ERBB2* [1]. HER2 was first functionally implicated in human breast cancer pathogenesis in 1987 when it was discovered that ERBB2 overexpression was a significant predictor of both overall survival (OS) and time to relapse [2]. ERBB2 is also overexpressed in subsets of patients with a variety of other cancers, including gastric, non-small cell lung, colon, bladder, and biliary cancers [1, 3–6].

Gastric cancer (GC) has unique HER2 immunostaining characteristics, with an incidence of up to 30% of intratumoral heterogeneity [7]. As such, the criteria for determining whether a tumor is HER2-positive differs between gastric and breast cancers [8]. HER2-positive GC is defined as HER2 immunohistochemistry (IHC) 3 + or 2 + /in situ hybridization (ISH) + (*ERBB2*/*CEP17* ≥ 2.0) and accounts for approximately 15% of GCs. Given the prevalence of HER2-postive GC, therapies targeting HER2 have been evaluated in this patient population.

In the ToGA trial, treatment with the anti-HER2 monoclonal antibody, trastuzumab, showed improved outcomes in combination with chemotherapy versus chemotherapy alone in patients with HER2-positive GC [9]. The results of this trial led to the widespread approval of trastuzumab for HER2-postive GC. Other HER2-targeting agents have been evaluated for the treatment of HER2-positive GC but have failed to show survival benefits in patients with GC despite demonstrating significant activities in HER2-positive breast cancer. This indicates unique challenges for the development of anti-HER2 treatment for HER2-positive GC.

The concept of antibody–drug conjugates (ADCs) was developed to combine the advantages of highly specific monoclonal antibodies with the cytotoxic effects of chemotherapeutic drugs to deliver a therapeutic amount of a cytotoxic drug directly to the target tissue with lower systemic toxicity [10]. While this concept is attractive as a potential strategy for the treatment of targetable tumors, the development of a successful ADC drug has some challenges. As an example, T-DM1, an ADC of trastuzumab and the cytotoxic microtubule inhibitor DM1, failed to show superiority to taxane in previously treated, HER2-positive advanced GC in the phase 2/3 GATSBY trial [11]. The heterogenous nature of HER2 expression in GC may have affected the activity of T-DM1, which does not have a bystander antitumor effect [12]. However, an ADC with a bystander antitumor effect may be the breakthrough needed to develop a successful ADC treatment for HER2-positive GC.

Trastuzumab deruxtecan (T-DXd, DS-8201) is an anti-HER2 human monoclonal IgG1 antibody, with the same amino acid sequence as trastuzumab, covalently linked to deruxtecan, which consists of an enzymatically cleavable peptide-based linker and a novel topoisomerase I inhibitor exatecan derivative (DXd), as its released payload [10, 13]. It was first approved for the treatment of patients with unresectable or metastatic HER2-positive breast cancer by the Food and Drug Administration (FDA) in the US (December 2019) [14], followed by approval in Japan (March 2020) for the same indication. It was recently approved in Japan (September 2020) as the first ADC in the world for the treatment of patients with HER2-positive unresectable or metastatic gastric or gastroesophageal junction (GEJ) cancer that progressed on cancer chemotherapy [15]. The current Japanese treatment guidelines recommend T-DXd as third- or later-line treatment for previously treated HER2-positive [16]. Very recently in the US, T-DXd has been approved for the treatment of adult patients with locally advanced or metastatic HER2-positive gastric or GEJ adenocarcinoma who have received a prior trastuzumab-based regimen [17]. It is also approved in the EU for the treatment of HER2-positive metastatic breast cancer [18].

In this review, we discuss the early development and characteristics of T-DXd, including its mechanism of action, structure, and preclinical findings. We then review the clinical findings of T-DXd in patients with HER2-positive GC, focusing on safety and tolerability. Treatment management for patients who experience T-DXd-related ILD and other adverse events (AEs) is discussed, and recommendations are provided based on the authors’ experience. Finally, future perspectives for T-DXd treatment in clinical practice, including therapeutic evaluations in ongoing clinical trials and author opinions on important future research, are discussed.

## Functional mechanism of ADCs

ADC technology delivers a cytotoxic payload linked to a highly specific targeting antibody to target cells in a concentrated fashion. After administration, the ADC is theorized to circulate until it locates and binds to its target antigen or is catabolized. Binding triggers receptor-mediated internalization and, once internalized, the payload takes action on the cell. In general, the development of ADCs faces the following three challenges: (1) payloads are limited as there are few agents with the subnanomolar range of cytotoxic activity required for ADC payload candidates; (2) drug linker instability may result in the release of the drug into the circulation; and (3) there is a need to achieve higher drug loading (i.e., an increased number of payload molecules per antibody molecule) as this is expected to lead to better efficacy [10]. T-DXd was created as a novel ADC that could overcome these challenges using advanced ADC technology.

## T-DXd

### Discovery, characteristics, and structure

Exatecan mesylate (DX-8951f) is a topoisomerase I inhibitor that was in development for the treatment of cancer starting in 1994. Clinical trials evaluated DX-8951f in patients with cancer [19, 20]; however, DX-8951f plus gemcitabine did not demonstrate any survival or clinical benefit over the comparator (gemcitabine) in a phase 3 study [20], so development was discontinued. Researchers then turned their attention to investigating the potential of a DX-8951f derivative, DXd, as a potential payload for trastuzumab using ADC technology. It was hoped that targeted delivery would overcome the efficacy and toxicity issues of DX-8951f that resulted from insufficient local concentrations of the drug.

Using a DNA topoisomerase I inhibition assay, DXd was reported to have a potency ten fold higher than SN-38, the active form of the prodrug irinotecan that is used to treat multiple solid tumors [13, 21–23]. This potency indicates that DXd would be an ideal payload candidate for ADC. Another important consideration in ADC development is the stability of the linker in plasma, which is important to minimize systemic toxicity. To address this, DXd was conjugated to cysteine residues on trastuzumab using an enzymatically cleavable peptide-based linker [24]. The drug-to-antibody ratio (DAR) of T-DXd is approximately 8, the highest of any currently approved ADC and the theoretical maximum achievable with conventional interchain cysteine conjugation [10]. For comparison, T-DM1 has a DAR of 3.5 [13].

Preclinical studies showed promising activity for T-DXd. The drug exhibited an antitumor effect in cells with high HER2 expression that was similar to T-DM1 and also had a significantly higher antitumor effect in cells with low HER2 expression [13].

### Mechanism of action

**Fig. 1:**
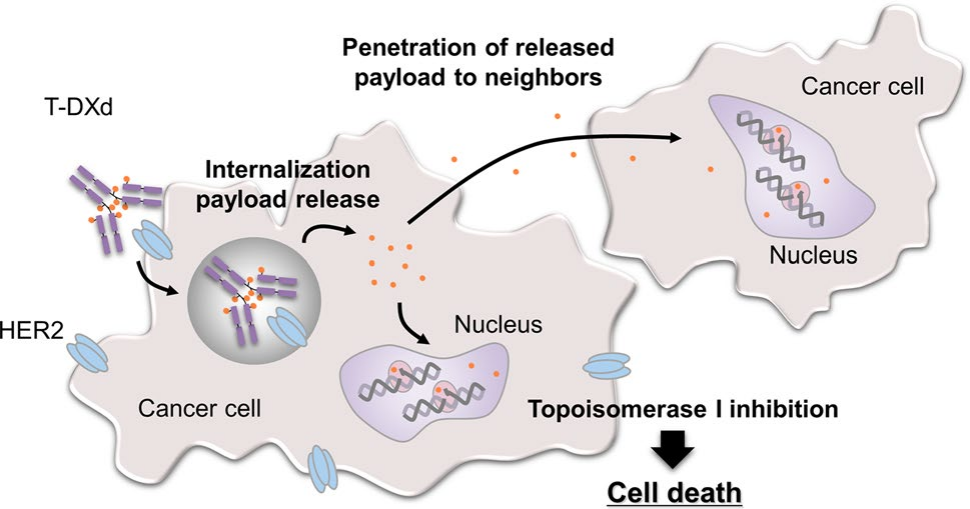
Antibody–drug conjugate functional mechanism and bystander antitumor effect of trastuzumab deruxtecan. *T-DXd* trastuzumab deruxtecan; *HER2* human epidermal growth factor receptor 2

T-DXd is expected to target the same antigen as trastuzumab but to have the added benefit of targeted delivery of the cytotoxic payload. The mechanism of action of T-DXd is bystander antitumor effect, which occurs when the cytotoxic payload is released in the tumor cells, diffuses across membranes (due to the high membrane permeability of the payload), and then enters and kills neighboring tumor cells (Fig. 1) [10, 13, 25]. This feature is particularly desirable when targeting tumors with heterogenous expression of the targeted antigen, as is the case for HER2-positive GC. Preclinical studies have shown that DXd is highly membrane permeable, and a bystander antitumor effect was demonstrated for T-DXd but not for T-DM1 [26]. Of note, the bystander antitumor effect by DXd was only observed in cells neighboring HER2-positive cells, and this may have been attributable to the short systemic half-life of the payload (1.37 h in animal models) [26, 27].

## Pharmacokinetic properties

The pharmacokinetics of T-DXd were evaluated in the dose escalation part of the open-label phase 1 study (DS8201-A-J101; NCT02564900) that included patients with breast, gastric, or gastroesophageal cancer with varying HER2 status that was refractory to standard therapy [28]. T-DXd showed a non-linear pharmacokinetic profile and the half-life of T-DXd increased at higher doses; drug exposure increased more than the dose ratio at doses above 3.2 mg/kg. Importantly, the pharmacokinetic analysis in this study showed there was no significant difference between the serum concentration of T-DXd and that of the antibody itself; thus, low systemic exposure of DXd was observed. The findings suggest that the linker of T-DXd is stable in the circulation. This observation is supported by a report of favorable in vitro stability of T-DXd in human plasma [13]. Based on the phase 1 analyses of pharmacokinetics, efficacy, and safety, a recommended dose of 6.4 mg/kg every 3 weeks was set for patients with GC.

T-DXd levels are reduced in the circulation due to degradation, internalization into target cells, and non-specific uptake by cells belonging to the reticuloendothelial system, such as macrophages and monocytes, that have the capability of phagocytosing foreign substances. DXd undergoes hepatobiliary excretion [29]; therefore, consideration may need to be given to patients with hepatic impairment. Currently, there are no dose adjustment recommendations for patients with mild or moderate hepatic impairment; however, the prescribing information for patients states that patients with moderate hepatic impairment should be closely monitored for increased toxicities related to DXd [29, 30]. There are no data yet to guide recommendations in patients with severe hepatic impairment, indicating a possible need to address the usage of T-DXd in these patients in future studies. In clinical studies, the impact of AUC_0–17 days_ on coadministration of CYP3A and/or organic anion transporting polypeptide inhibitors with T-DXd has not been clinically meaningful [30].

## Therapeutic efficacy

### Phase 1 and 2 gastric cancer trials

Data from the phase 1 DS8201 A-J101 study [31] and the phase 2 DESTINY-Gastric01 study (NCT03329690) [25] established the dose and efficacy of T-DXd. The DESTINY-Gastric01, for patients with HER2-positive gastric or GEJ cancer who were previously treated with ≥ 2 lines of therapy, including trastuzumab, met its primary endpoint of significantly improved objective response rate for T-DXd versus physician’s choice (PC) treatment (51% versus 14%, respectively; *P* < 0.001) [25]. The median duration of confirmed objective response for T-DXd was 11.3 months, which was notable considering that the duration of response for first-line trastuzumab plus chemotherapy treatment was reported as 6.9 months [9]. These well-known trials are reviewed in a recent publication by Kotani and Shitara [32].

## Safety and tolerability

### Phase 1 and 2 gastric cancer trials

The safety profile of T-DXd in patients with GC is generally manageable; safety and tolerability results for the T-DXd phase 1 and phase 2 clinical trials are summarized below [25, 31]. At least one treatment-emergent adverse event (TEAE) was reported for all patients in both trials. TEAEs that occurred in ≥ 20% of patients in the DESTINY-Gastric01 phase 2 trial are shown in Table 1. The majority of the most common AEs were gastrointestinal or hematological in nature, and the most common AEs and their incidences were similar between the two trials, with the exception of the incidence of neutrophil count decreased (all grades), which was higher in the phase 2 trial (63%) compared with the phase 1 trial (27%). Approximately half of the patients in the phase 1 trial were treated with 5.4 mg/kg T-DXd and half with 6.4 mg/kg, whereas all patients in the phase 2 trial were treated with 6.4 mg/kg; this dosing difference may account for the difference in the incidence of neutrophil count decreased. Of the AEs occurring in ≥ 20% of patients treated with T-DXd, the most frequent grade ≥ 3 events were hematological; anemia (30% versus 38%, respectively), neutrophil count decreased (20% versus 51%, respectively), and white blood cell count decreased (16% versus 21%, respectively). In the phase 1 study, there were four cases of pneumonitis (grade 2, *n* = 3 [7%]; grade 3, *n* = 1 [2%]), all were evaluated by an independent adjudication committee, which determined that three (7%) patients had ILD related to treatment and one had grade 3 ILD that was not related to treatment (unpublished data; data cutoff: 1 February 2019). The phase 2 study reported 12 (9.6%) patients with ILD or pneumonitis related to treatment. Regarding deaths, the phase 1 study reported two (pneumonia and disease progression), neither of which was considered treatment drug-related; the phase 2 study reported one death due to pneumonia that was considered treatment drug-related.

**Table 1:** Adverse events occurring in at least 20% of the patients treated with trastuzumab deruxtecan.

Preferred term	Trastuzumab deruxtecan (*N* = 125)			Physician’s choice of chemotherapy (*N* = 62)		
	Any grade No. of patients (percent)	Grade 3	Grade 4	Any grade	Grade 3	Grade 4
Nausea	79 (63)	6 (5)	0	29 (47)	1 (2)	0
Neutrophil count decreased^a^	79 (63)	48 (38)	16 (13)	22 (35)	10 (16)	5 (8)
Decreased appetite	75 (60)	21 (17)	0	28 (45)	8 (13)	0
Anemia^b^	72 (58)	47 (38)	0	19 (31)	13 (21)	1 (2)
Platelet count decreased^c^	49 (39)	12 (10)	2 (2)	4 (6)	1 (2)	1 (2)
White cell count decreased^d^	47 (38)	26 (21)	0	22 (35)	5 (8)	2 (3)
Malaise	43 (34)	1 (1)	0	10 (16)	0	0
Diarrhea	40 (32)	3 (2)	0	20 (32)	1 (2)	0
Vomiting	33 (26)	0	0	5 (8)	0	0
Constipation	30 (24)	0	0	14 (23)	0	0
Pyrexia	30 (24)	0	0	10 (16)	0	0
Alopecia	28 (22)	0	0	9 (15)	0	0
Fatigue	27 (22)	9 (7)	0	15 (24)	2 (3)	0
Lymphocyte count decreased^e^	27 (22)	8 (6)	6 (5)	2 (3)	0	1 (2)

From Shitara et al. [25]. © (2020) Massachusetts Medical Society. Reprinted with permission

No additional adverse events during the trial were observed in at least 20% of the patients receiving physician’s choice of chemotherapy

^a^This category includes the preferred terms neutrophil count decreased and neutropenia

^b^This category includes the preferred terms hematocrit decreased, hemoglobin decreased, red-cell count decreased, and anemia

^c^This category includes the preferred terms platelet count decreased and thrombocytopenia

^d^This category includes the preferred terms white-cell count decreased and leukopenia

^e^This category includes the preferred terms lymphocyte count decreased and lymphopenia

TEAEs that led to dose reductions were reported in 16% and 32% of patients in the phase 1 and phase 2 trials, respectively. It should be noted that in the phase 2 study, TEAEs that led to dose reduction were similar between the T-DXd and PC treatment groups (32% and 34%, respectively).

In the DESTINY-Gastric01 trial, there was a higher incidence of grade ≥ 3 hematological AEs in patients treated with T-DXd compared with those treated according to the PC [25]. Additionally, the T-DXd group had a higher rate of TEAEs associated with treatment discontinuation (15% versus 6%) and treatment interruptions (62% versus 37%) compared with the PC group, indicating a need for physician awareness related to the management of TEAEs in patients receiving T-DXd.

Cardiotoxicity is known to be a serious side effect in patients with breast cancer who are treated with HER2-targeted therapies including trastuzumab [33–35]; however, the incidence of cardiotoxicity in patients with gastric or breast cancer treated with T-DXd appears to be low and was not observed in the DESTINY-Gastric01 trial [9, 31].

### Interstitial lung disease

HER2-targeted therapies, including T-DXd, are also associated with a risk of ILD [29, 36]; specific guidance for managing ILD during treatment has been established for use in all T-DXd studies. Historically, the incidence of ILD is higher in Japan than in non-Japan [37]. An analysis was performed to identify potential risk factors of T-DXd for ILD using available data from the DS8201-A-J101 study and DESTINY-Breast01 trial (HER2-positive breast cancer; NCT03248492) [38, 39]. Analysis of the pooled data showed that 80.6% of patients had breast cancer and 43% of patients were from Japan. This analysis revealed a 16.8% adjudicated drug-related incidence of ILD across the two studies. The majority was grade 1, 4.8% or grade 2, 8.3%. Grade ≥ 3 was 3.7% [38, 39]. The incidence was numerically lower in patients with non-breast cancer versus breast cancer (11.4% versus 18.1%) and in patients with HER2-positive breast cancer who were treated with a lower dose (5.4 mg/kg, 13.7%; 6.4 mg/kg, 22.8%). However, the only potential risk factor associated with treatment-related ILD in both univariate and multivariate analysis was residence in Japan (univariate analysis: Japan versus countries other than Japan: odds ratio [OR], 2.6; 95% CI 1.6–4.1; *P* < 0.001; multivariate logistic regression analysis: OR, 3.6; 95% CI 2.1–6.1; *P* < 0.001; multivariate Cox regression analysis: OR, 2.5; 95% CI 1.6–4.0; *P* < 0.001) [39].

Rates of ILD according to grade are shown in Table 2 for each of the DESTINY phase 2 clinical studies. The median time to onset for patients who experience ILD is much longer for patients with breast cancer (median, 194 days; range 42–535 days) than for other cancers (gastric: median, 84.5 days; range 36–638 days; lung: median, 86 days; range 41–255 days; colorectal: median, 80 days; range 22–132 days) [25, 40–42]. The prior treatment lines and T-DXd treatment duration might be related to the reason why median time to onset could be different across tumor types.

**Table 2:** Incidence of interstitial lung disease in clinical studies of trastuzumab deruxtecan

Trial name	T-DXd (mg/kg)	No. of patients	Grade 1	Grade 2	Grade 3	Grade 4	Grade 5	Total	ClinicalTrials.gov Identifier	References
DESTINY–Gastric01	6.4	125	3 (2.4)	6 (4.8)	2 (1.6)	1 (0.8)	0	12 (9.6)	NCT03329690	[25]
DESTINY–Breast 01	5.4	184	20 (10.9)^a^		1 (0.5)	0	4 (2.2)	25 (13.6)	NCT03248492	[40]
DESTINY–Lung 01	6.4	42	0^b^	5 (11.9)	0	0	0	5 (11.9)	NCT03505710	[41]
DESTINY–CRC01	6.4	78	0	2 (2.6)	1 (1.3)	0	2 (2.6)	5 (6.4)	NCT03384940	[42]

^a^Grade 1 and 2 combined

^b^One case of potential grade 1 interstitial lung disease was pending adjudication at the time these data were collected

The mechanism by which T-DXd is involved in the development of ILD is currently unknown. Preclinical studies in monkeys found that high level of T-DXd exposure resulted in the development of a similar pathology to what was observed in the treatment-associated ILD/pneumonitis in humans [43]. In this model, T-DXd was detected in HER2-negative lung alveolar macrophages, but not in the airway epithelium including HER2-positive bronchial and bronchiolar epithelial cells. Macrophages expressed cathepsin B, one of the enzymes capable of linker cleavage of T-DXd [24, 44]. Together, these findings in this monkey toxicology model may suggest a possible involvement of target independent T-DXd uptake by alveolar macrophages potentially followed by linker cleavage and release of free DXd.

### Nausea

Nausea is a frequently reported TEAE with T-DXd treatment. Nausea of any grade was reported for 63% of patients in the DESTINY-Gastric01 trial and 78% of patients in the DESTINY-Breast01 trial; the respective incidences of grade ≥ 3 nausea were 5% and 8% [25, 40]. These results indicate that the management of nausea is important during T-DXd treatment.

## Management of T-DXd treatment in clinical practice

### Interstitial lung disease

**Fig. 2:**
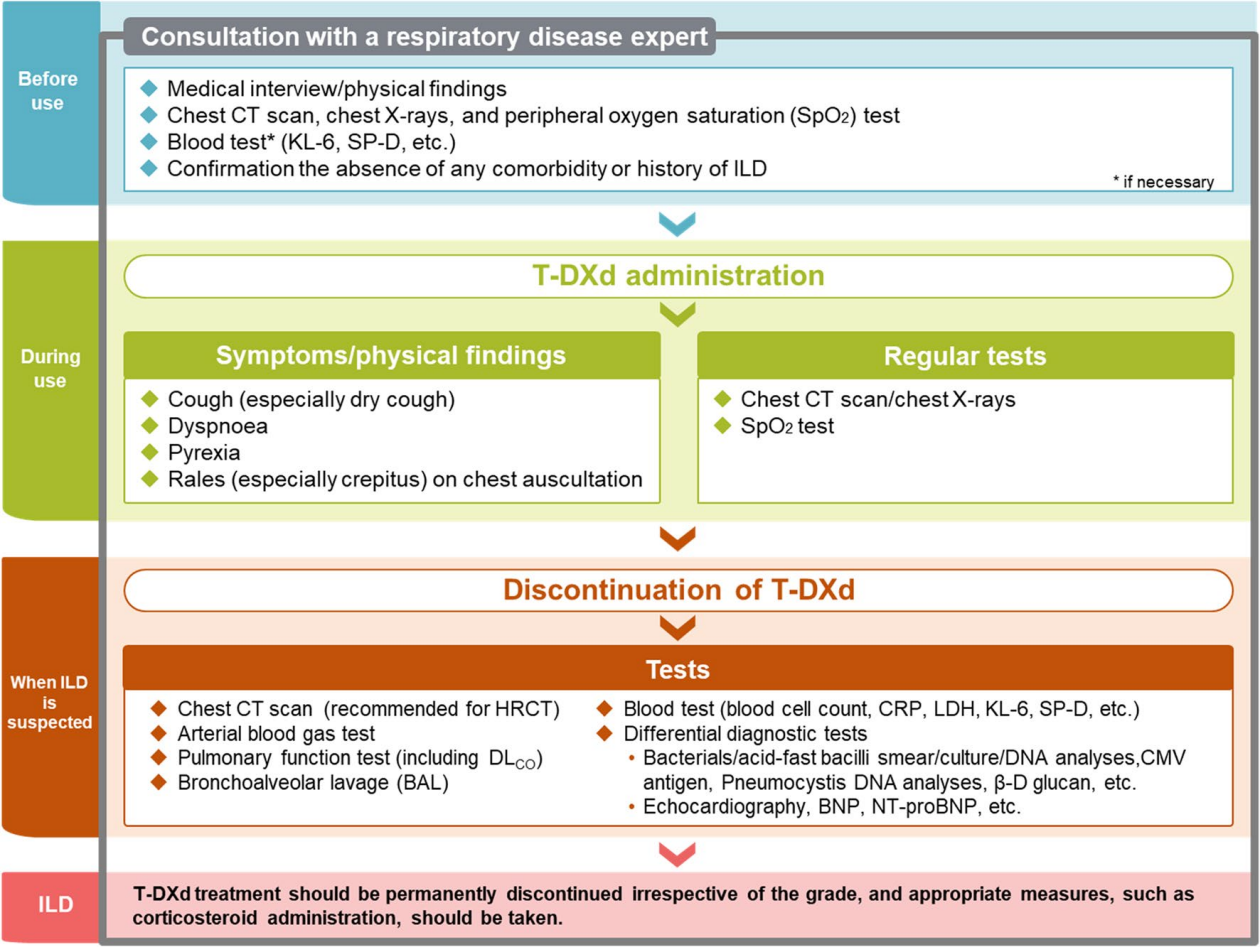
Management flowchart of T-DXd-induced interstitial lung disease. *BNP* brain natriuretic peptide; *CMV* cytomegalovirus; *CRP* c-reactive protein; *CT* computed tomography; *DL*_*CO*_ diffusing capacity of the lung carbon monoxide; *HRCT* high resolution computed tomography; *ILD* interstitial lung disease, *KL-6* Krebs von den Lungen-6; *LDH* lactate dehydrogenase; *SP-D* pulmonary surfactant protein-D; *T-DXd* trastuzumab deruxtecan

A guide for the appropriate use of T-DXd in Japanese clinical practice, positioned as part of a risk management plan [45] and aligned with the Pharmaceuticals and Medical Devices Agency has been prepared by Daiichi Sankyo Co., Ltd [46]. One of the approaches to the management of T-DXd-related ILD safety that is included in the guide by Daiichi Sankyo Co., Ltd., was developed with reference to the guide published by Kubo et al. [37] and is shown in Fig. 2. The guide outlines the recommended approach for ILD monitoring and diagnosis in clinical practice and includes monitoring for initial symptoms of ILD and performing regular chest computed tomography scans, chest X-rays, and peripheral oxygen saturation testing for early detection and intervention, in collaboration with a respiratory disease expert. The guide and the T-DXd package insert [47] state that physicians should confirm the absence of any comorbidity or history of ILD prior to the initiation of the T-DXd treatment, and T-DXd should be permanently discontinued in patients with ILD, irrespective of the grade, and appropriate measures, such as corticosteroid administration, should be taken. This statement is stricter than those used in the DESTINY-Gastric01 trial [25]. Of note, in the DESTINY-Gastric01 trial, patients with GC were excluded if they had or were suspected to have interstitial lung disease/pneumonitis, or if they had a history of noninfectious interstitial lung disease/pneumonitis that had been treated with steroids. The authors recommend that the flowchart shown in Fig. 2 be used as a guide for the appropriate use of T-DXd and management of T-DXd-related ILD in clinical practice.

### Nausea and vomiting

As previously mentioned, nausea and vomiting are commonly reported with T-DXd treatment (nausea any grade, 63–78%; nausea grade ≥ 3, 5–8%; vomiting any grade, 26–46%; vomiting grade ≥ 3, 0–4%) [25, 40], which highlights a need for effective management. One potential strategy is prophylactic administration of antiemetic medications; however, the frequency at which this management strategy is used is unknown, and there are no data available outlining whether this strategy is preventative in patients treated with T-DXd. The National Comprehensive Cancer Network (NCCN) recommends treatment of moderate nausea/vomiting; recommended treatments include dexamethasone, serotonin receptor (5-HT_3_) antagonists, and/or aprepitant [48]. Specific recommendations differ, depending on whether the nausea is acute or delayed. In general, the authors agree that these recommendations are appropriate.

### Hematological toxicity

Hematological TEAEs are also commonly reported in patients receiving T-DXd [25, 40–42]. To date, most hematological TEAEs reported for T-DXd in GC clinical trials were manageable with appropriate dose modification and supportive treatment, with few leading to T-DXd discontinuation [25]. Granulocyte-colony stimulating factor (G-CSF) is widely used to treat neutropenia, as it can promote the activation, proliferation, and differentiation of myeloid precursor cells [49]. The NCCN guidelines recommend G-CSF treatment for prophylaxis of febrile neutropenia based on patient risk factors [50]. The authors recommend dose reduction, interruption, or discontinuation in the event of hematological TEAEs and visits at day 1 of each treatment cycle for regular blood testing. In addition, the authors recommend visits at days 8 and 15 of treatment cycle 1 when possible, as this was required in the DESTINY-Gastric01 trial. If needed, treatment with G-CSF, antibiotics, or blood transfusion should be considered.

## Future perspectives

### Ongoing studies for HER2-positive gastric cancer

There are several ongoing clinical studies to further evaluate the efficacy and safety of T-DXd, either alone or in combination with other drugs, for the treatment of HER2-positive (IHC 3 + or IHC 2 + /ISH +) GC. Current ongoing trials include the phase 2 DESTINY-Gastric02 study (NCT04014075; T-DXd; second line) [51], the phase 1b/2 DESTINY-Gastric03 study (NCT04379596; T-DXd monotherapy or T-DXd + chemotherapy and/or durvalumab; Part 1 second or later line; Part 2 first line) [52], and the phase 3 DESTINY-Gastric04 study (NCT04704934; T-DXd or ramucirumab + paclitaxel; second line). It is hoped that the results from these ongoing clinical trials will help pave the way for T-DXd in earlier lines of treatment as a single agent or in combination with other agents. Along with efforts to advance the treatment of HER2-postive GC, it is important to develop more effective T-DXd treatment strategies for patients with HER2-low tumors, as these patients are not included in the trials evaluating T-DXd for HER2-positive GC.

### Further studies for the treatment of HER2-low tumors

The tumor expression level of HER2 has been shown to have an impact on the efficacy of T-DXd. In GC, response rates are reported to be higher in patients with tumors having a higher HER2 expression (IHC 3 +) compared with patients whose tumors have a lower expression (IHC 2 + and ISH +) with respective objective response rates of 58% and 29%; however, concrete conclusions cannot be drawn as the sample size for this study was small [25]. Although, to date, there are no data to suggest why this is the case, the authors speculate that the bystander antitumor effect is greater with higher expression of HER2 because of longer retention effect. Future studies correlating HER2 expression to the bystander antitumor effect of T-DXd are warranted.

Results from patients in exploratory cohorts in the DESTINY-Gastric01 trial who were confirmed to have HER2-low tumors (IHC 2 + /ISH- [cohort 1] or IHC 1 + [cohort 2]) demonstrated that T-DXd had some anti-tumor activity [53]. Cohort 1 had a confirmed objective response rate of 26.3%, and a median PFS and OS of 4.4 and 7.8 months, respectively; cohort 2 had a confirmed objective response rate of 9.5%, and a median PFS and OS of 2.8 and 8.5 months, respectively. In patients with HER2-low breast cancer, T-DXd demonstrated promising preliminary anti-tumor activity, with a confirmed objective response rate of 37%, median PFS of 11.1 months, and median OS of 29.4 months [54].

Taken together, these data point to a need for more rigorous and sensitive HER2 testing methods to identify HER2-low patients who might benefit from T-DXd treatment. Additionally, variable HER2 expression in primary versus metastatic tumors [55] and the potential for HER2 expression levels to change with treatment, particularly with currently approved HER2-targeted therapies [56], supports the need for testing not only at diagnosis, but also when treatment failure occurs.

Future studies analyzing HER2 expression in fresh biopsy and circulating tumor DNA will help shed light on the relationship between HER2 expression and T-DXd efficacy and the bystander antitumor effect, possibly leading to improved patient selection. These analyses may elucidate the molecular mechanisms of primary and acquired T-DXd resistance in GC. Additionally, research to identify biomarkers that can optimize patient selection, identify early response signals, and inform potential combination regimens is expected to improve treatment success.

Clinical practice around treatment strategies with T-DXd is expected to evolve with clinical experience and some modifications to treatment may be needed. As has been the case with the treatment of HER2-positive breast cancer, it may be necessary to modify the treatment of HER2-positive GC based on subtype (e.g., Epstein–Barr virus-positive, microsatellite instability, genomically stable, or chromosomal instability [57]) from the first treatment to the second- and third-line treatments. Combination therapy including T-DXd and a cytotoxic drug, such as S-1 + leucovorin + T-DXd, may be effective in patients with GC, and it is anticipated that future clinical trials will explore novel treatment combinations that include T-DXd. Finally, the effects of T-DXd treatment in patients without targeted lesions, patients with a poor Eastern Cooperative Oncology Group performance status (ECOG PS), and patients with peritoneal dissemination and severe ascites who were not included in previous clinical trials will become clear as real-world clinical data become available.

## Conclusion

Although several HER2-targeted therapies have been investigated in HER2-positive advanced GC, until recently, trastuzumab was the only approved anti-HER2 agent. T-DXd was developed to overcome GC-specific challenges for HER2-targeted therapy, which might have been achieved largely through a bystander antitumor effect and a high DAR. T-DXd is the first HER2-targeting ADC that has demonstrated a superior response rate and survival benefit over standard chemotherapy. Overall, T-DXd has a manageable safety profile; however, patients should be carefully monitored for ILD in clinical practice. The recommendations in this review are intended to assist physicians with T-DXd treatment management.
